# Differences in Mismatch Responses to Vowels and Musical Intervals: MEG Evidence

**DOI:** 10.1371/journal.pone.0076758

**Published:** 2013-10-15

**Authors:** Elika Bergelson, Michael Shvartsman, William J. Idsardi

**Affiliations:** 1 Department of Psychology, University of Pennsylvania, Philadelphia, Pennsylvania, United States of America; 2 Department of Psychology, University of Michigan, Ann Arbor, Michigan, United States of America; 3 Department of Linguistics, University of Maryland, College Park, Maryland, United States of America; Northwestern University, United States of America

## Abstract

We investigated the electrophysiological response to matched two-formant vowels and two-note musical intervals, with the goal of examining whether music is processed differently from language in early cortical responses. Using magnetoencephalography (MEG), we compared the mismatch-response (MMN/MMF, an early, pre-attentive difference-detector occurring approximately 200 ms post-onset) to musical intervals and vowels composed of matched frequencies. Participants heard blocks of two stimuli in a passive oddball paradigm in one of three conditions: sine waves, piano tones and vowels. In each condition, participants heard two-formant vowels or musical intervals whose frequencies were 11, 12, or 24 semitones apart. In music, 12 semitones and 24 semitones are perceived as highly similar intervals (one and two octaves, respectively), while in speech 12 semitones and 11 semitones formant separations are perceived as highly similar (both variants of the vowel in ‘cut’). Our results indicate that the MMN response mirrors the perceptual one: larger MMNs were elicited for the 12–11 pairing in the music conditions than in the language condition; conversely, larger MMNs were elicited to the 12–24 pairing in the language condition that in the music conditions, suggesting that within 250 ms of hearing complex auditory stimuli, the neural computation of similarity, just as the behavioral one, differs significantly depending on whether the context is music or speech.

## Introduction

Music and language understanding both require the listener to abstract over various kinds of information contained in the acoustic stream. When understanding an uttered sentence we can attend primarily to the meaning of the sentence and thereby abstract over speaker, gender, volume, emotion, dialect, and other speaker and context specific variables; similarly, when listening to a musical phrase we can focus on the melody and abstract over timbre, volume, emotion, pitch, and register. That is, while listeners can certainly discriminate various acoustic properties in the language and speech input, they can also abstract over them when recognizing words or musical elements. Thus, on a coarse-grained scale many parallels exist across these domains, but nevertheless there has been much debate about whether music and language processing share cognitive mechanisms [1], [2].

Narrowing our focus to the perceptual organization of the two systems, we find differences in the perception of one of the smallest complex units of language and music, i.e. simultaneously sounded notes, which reliably elicit holistic perceptions as vowels, and as musical chords. Judgments of vowel similarity follow acoustic measures fairly directly: the more acoustically different two vowels are, the less similar they are judged to be [3]. Musical similarity is more complicated, with consonance and similarity judgments in music following a non-linear relation [4]–[7]. We explore this reported difference directly, comparing the processing of musical and linguistic stimuli (piano tones, sine waves, and vowels) that are carefully matched in frequency to examine how a neural measure of similarity may vary across these domains.

We created stimuli that were maximally acoustically similar, so that neurological differences could be more clearly attributed to stimulus type (sinusoid, piano, language) rather than acoustic structure differences. To this end we compared two-formant vowels and simultaneously sounded two-note musical intervals; each stimulus was made up of two primary frequencies instantiated either as a vowel or musical interval. While vowels in natural speech consist of many formants, humans are readily able to interpret vowels instantiated with two formants [8]; our musical intervals are instances of the simplest form of harmony.

### Similarity in Vowel Space

Vowels are commonly represented on a quadrilateral plotting the first formant (i.e. first resonant frequency) on the ordinate and the second formant on the abscissa. Vowels themselves are often indicated by ellipses since phonetic measurement showing the precise location of a vowel varies by instance within and across speakers (e.g., [9]). Perceptually, vowels that are close in acoustic space are heard as more similar than vowels which are further apart. Though the vowel system diverges from absolute linearity in some ways (e.g., [10]), perceptual similarity generally correlates with acoustic proximity.

For example, early work querying the correlation in the perceptual and acoustic space of 11 American English vowels found very high correspondence between the physical properties of the stimuli and adults' judgments of similarity [3]. The study also found that first and second formant frequencies were critical factors in determining vowel perception.

Thus, the previous literature demonstrates that the vowel acoustic space is somewhat cluttered [9], and that acoustic proximity generally leads to perceptual similarity [3].

### Similarity in Music Space

In contrast, the location of musical intervals in acoustic space is much more precise, and there are sharp non-linearities between intervals whose component sounds are proximally close acoustically. Research investigating adults' similarity judgment of simultaneously sounded intervals finds that simple-tone intervals (i.e. those lacking additional harmonics) are differentiated in terms of interval width but that the scale seems to be curvi-linear [7].

Other work on interval similarity has compared differences in consonance. Taking as a linking hypothesis that musical intervals with larger consonance differences will be judged more perceptually different, we can consider similarity in consonance to be a proxy for the sort of ‘simple’ similarity used in vowel perception. Across many studies, we find octaves, perfect fourths and perfect fifths to be very consonant, while major/minor sevenths and seconds sound the most dissonant [11]; this perceptual consonance/dissonance difference maps onto brain oscillation patterns in humans and monkeys as well [12]. Thus, intervals that differ by just one semitone, e.g. a major seventh and perfect octave, wind up on opposite ends of the consonance spectrum [4]. Moreover, beyond the octave, we find that a two octave interval is perceived as quite close in consonance to a single octave, while nearby semitone differences are perceived as quite different [5]. There are varying ideas about how the musical perceptual space may be organized, all of which try to specify the details of the nonlinearity (for a compelling account, see [13]). Previous research has thus made evident that the music and vowel acoustic spaces are not perceptually organized in the same way as indexed by behavioral measures; neural measures remain an open question.

### Mismatch Negativity (MMN)

To examine the domain-specificity in perceptual organization across music and language neurally, we used magnetoencephalography (MEG) to measure the mismatch negativity response (MMN), a neural difference detector (see [14] for a review). The MMN is a robust, automatic, pre-attentive, early response (around 150 ms to 250 ms post-stimulus onset) to (e.g. auditory) deviants in a series of standards. Moreover, MMN amplitude tracks the size of the perceived difference between standards and deviants, and is thus a good tool for determining *how* different the A and B stimuli are perceived as being. MMN designs allow one to compare the brain's response to stimulus A as standard versus that same A as deviant: only the *role* of the stimulus within the design (e.g. in contrast to the B stimulus) modulates the electrophysiological response, rather than any acoustic property per se, as the design matches all other aspects.

While neurological research linking music and language is still sparse (see [2] for a comprehensive review), several previous studies inform the questions we examine here, and support the validity of using an MMN design make comparisons across acoustic domains.

Tervaniemi and colleagues, in a within-participants oddball MMN design, presented participants with series of vowels and musical chords [15]. They found that the MMN to 3-note chord changes was larger than to vowel changes in the right hemisphere, while left hemisphere MMNs did not differ across domains. Our study varies from theirs in that we matched our speech and language stimuli in sound structure frequency. If modality is responsible for the hemisphere differences they observed, we should expect to find such differences as well. However, if the more broad acoustic differences between their stimuli caused hemispheric differences, we would not find such differences in our study.

Other work has examined the role of musical expertise in neural processing of music and language, finding differences between experts on certain measures, though not all [1], [16]–[18]. While we find this question compelling, we are not interested in the expertise variable here; rather, we queried an unselected group of individuals with normal hearing and typical language exposure.

### Primary Question of Interest

While the research described above informs global questions of what differences and similarities may exist in music and language processing, none makes an explicit and direct test of frequency-matched stimuli across the domains, as we do here.

**Figure 1:**
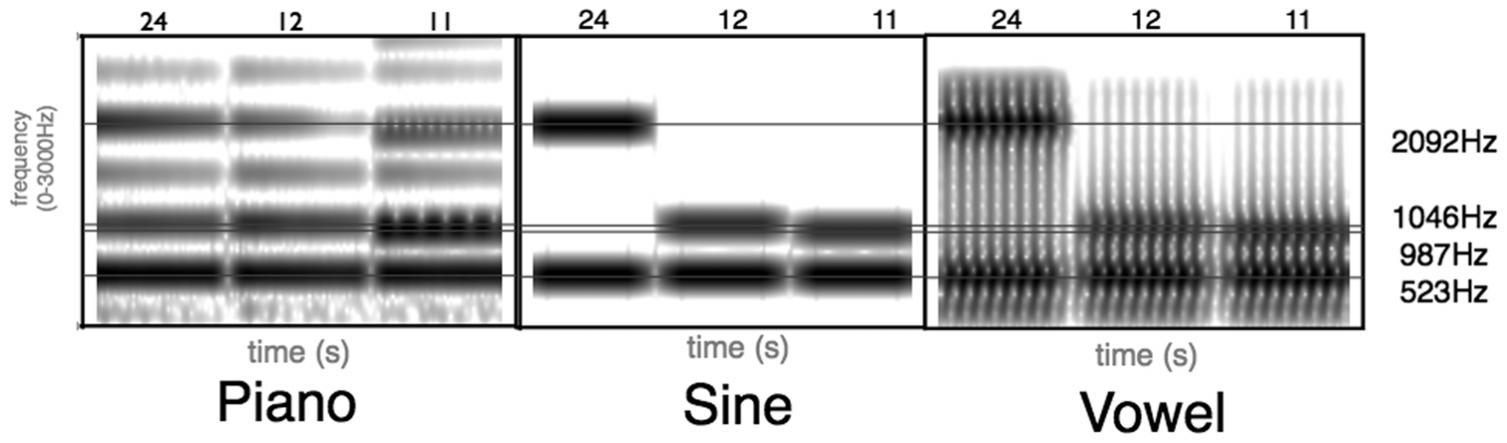
Spectrograms of experimental stimuli. Every interval consisted of two primary frequencies; all intervals had 523 as the lower frequency and 987, 1046, or 2092 as the upper frequency for 11, 12, and 24 respectively.

Specifically, we were interested in how the brain computes the similarity between stimuli with closely matched acoustic properties but from different content domains (music, language). That is, in comparing stimulus A and B within music and within language, is similarity computed independent of modality, based on purely acoustic features of A and B, or does the domain to which the stimuli belong matter, even in a response as early as the MMN? To test this we created stimuli made up of the same primary frequencies, instantiated either as vowels or musical intervals (see Figure 1 and details below). Specifically, the intervals we picked were 11 semitones (major 7^th^), 12 semitones (octave), and 24 semitones (double-octave), all having as their lower primary pitch C5 (523 Hz). These specific stimuli lead to an interesting dichotomy across domains: in music, 12 semitones and 11 semitones differ only by a single semitone but sound very different, while 12 semitones and 24 semitones differ by an entire octave, but sound very similar. In contrast, 11 semitones and 12 semitones instantiated as vowels sound like variants of the same mid-back vowel, while 24 semitones sounds like a different vowel (a front vowel).

Given the length of the experiment (60–90 minutes), participants were either in the 12–11 or 12–24 condition, with modality as a between-participants factor. For instance, if a participant was in the 12–11 vowel condition, they heard a block of stimuli where the 12 semitones was the standard with occasional 11 semitones deviants, and a block of stimuli where the 11 semitones was the standard with occasional 12 semitones deviants. Thus, across participants we are able to compare the response to deviant 12 semitones in the context of 11 semitones or 24 semitones, in music and in language.

### Predictions

If content domain matters, we expect the MMN in the 12–11 condition to be large in the music case and small in the vowel case, while in the 12–24 condition, the MMN should be large in the vowel case and small in the music case. Thus, we expect to find that the amplitude of the MMN will track the perceptual distance within the pairs given the content domain. That is, vowels should show a larger MMN to the comparison between 12 semitones and 24 semitones (which sound like different vowels) whereas the piano and sine wave tones should show larger MMN to the comparison between 12 semitones and 11 semitones (because 11 semitones is perceived as less similar to 12 semitones than 24 semitones is).

### Secondary Question of Interest

A common simplifying assumption made in auditory research is that sine waves are a good approximation of sounds with complicated spectral envelopes. However, since sine waves do not have any additional harmonics they may be impoverished stimuli compared to vowels or instrumental notes. In consonance assessment, pure tones seem to be treated somewhat differently from complex tones [5]. On the other hand, in absolute pitch possessors, note naming accuracy for piano tones and pure tones patterns together, separately from other timbres [19]. We test this issue directly by using simple pure sine waves, spectrally complex two-tone piano intervals, and two-formant vowels; we compared MMNs elicited by each of these types of stimuli in the 12–11 and 12–24 conditions described.

## Methods

### Ethics Statement

The involvement of human participants was approved by the IRB of the University of Maryland (College Park, Maryland, USA). All participants signed informed consent forms prior to the experiment. Participants received course participation credit or payment.

### Participants

55 adult volunteers participated in the study; 18 were excluded for various reasons (non-compliance, non-native English speakers, lack of identifiable response to the pre-test, equipment failure) leaving 37 usable participants (18 female; mean age 23.5 years). All participants gave written informed consent, had normal hearing, and had received standard exposure to English (for the vowel condition participants). Participants' level of musical expertise was not queried. Each session lasted for 60–90 min.

### Materials

**Figure 2:**
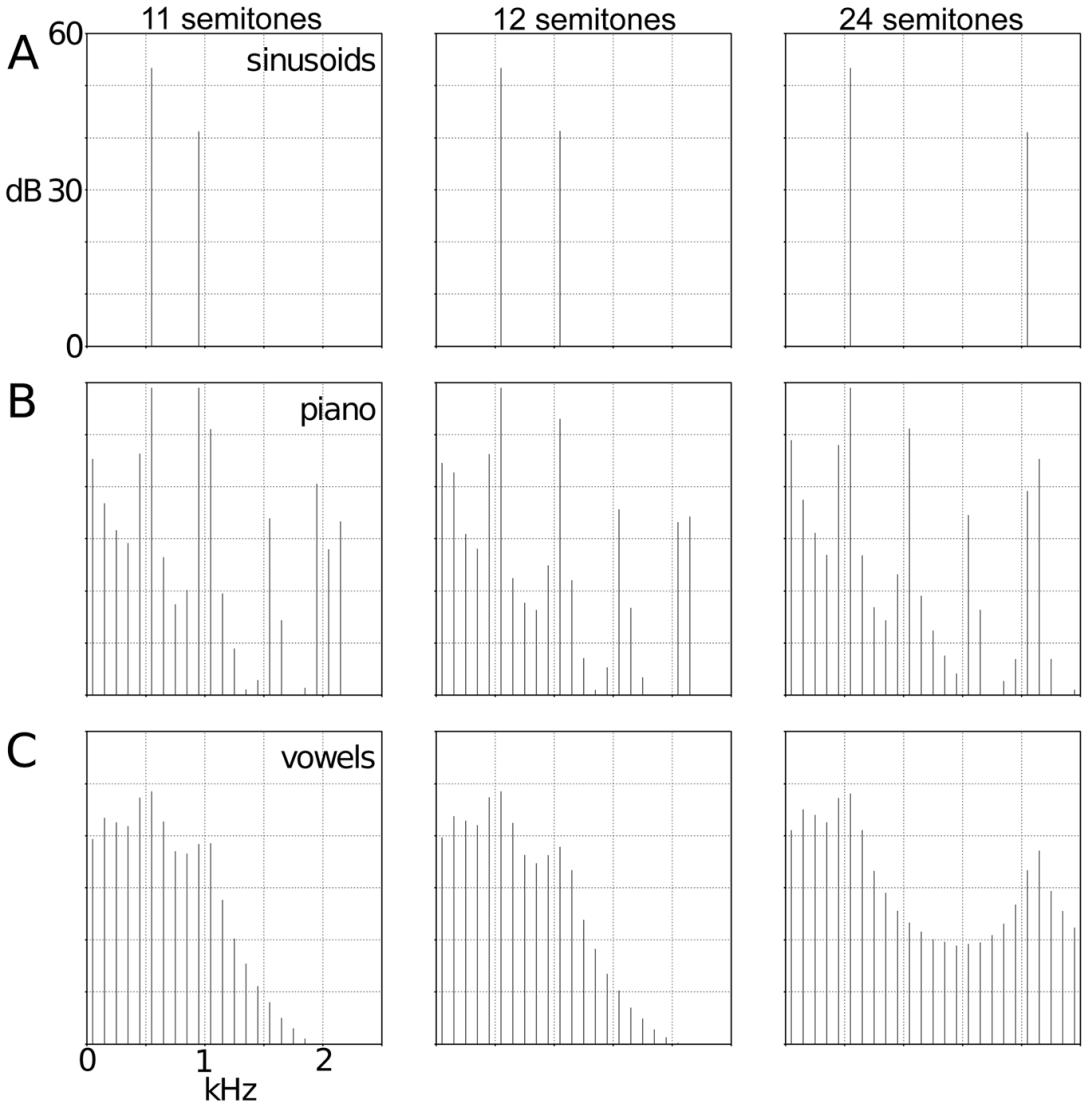
Line spectra of experimental stimuli. The primary frequencies from Figure 1 are evident in the peaks of the spectral responses for each item. The rows contain (top to bottom) sinusoids, piano tones and vowels. The columns contain (left to right) 11, 12 and 24 semitone intervals. Timbre differences amongst the stimuli are most evident in the spectral region between the two primary frequencies, especially in the two-octave interval, between 700 and 2000 Hz. There is essentially no harmonic energy in this region in the sinusoids, strong 2^nd^ and 3^rd^ harmonic responses for the piano chord (at 1046 and 1569 Hz respectively) and a smooth, quasi-parabolic contour in the vowel.

Auditory stimuli were of 3 types: pure sinusoids (S), piano tones (P), and synthesized two-formant vowels (V). The piano was chosen for two reasons. First, the piano provides a spectrally rich sound (see below), comparable to the richness produced by vowels. Second, because it is common for pianists to play chords (as opposed to clarinet players, for example) and we did not want to introduce the question of interpreting the sound as being produced by a single performer or by an ensemble of players. Each stimulus featured two primary simultaneous pitches that had identical lower frequency resonances (called “formants” in vowels; C5/523 Hz) and higher frequency resonances creating three intervals: 11 semitones (B5/987Hz), 12 semitones (C6/1046Hz), and 24 semitones (C7/2092Hz). Stimuli were digitized at 44.1 kHz, lasted 100 ms, including 20 ms on and off ramps, and had average amplitudes of 75 dB (see Fig. 1). The vowels were synthesized in Praat using the Klatt synthesizer, and were low-pass filtered at 3000Hz to remove higher formants. Vowels and piano tones are harmonically complex, with significant acoustic energy distributed across the spectrum (see Fig. 2). Vowels, in particular, are produced by overlaying a laryngeal source (produced by vocal fold vibrations) with a vocal tract filter (whose characteristics are controlled by the positioning of the tongue and lips). The laryngeal source provides the rich harmonic structure for the vowels, with acoustic energy concentrated at all integer multiples of the pitch frequency, in this experiment set to 100Hz. The physics of pianos and piano tuning also provides piano sounds with rich spectral content with acoustic energy across the spectrum. Figure 2 shows line spectra for the three kinds of sounds at the three different intervals. The sinusoids are the simplest, with almost all acoustic energy concentrated at the primary frequencies of interest. The piano and the vowel sounds are both much more harmonically complex, with acoustic energy distributed across the spectrum, differently in detail, providing the characteristically different timbres between the sounds. Phenomenologically, the sine wave and piano intervals sound like musical intervals. V11 semitones and V12 semitones sound like mid-back vowels (variants of [Λ], as in “cut”) whereas V24 semitones is perceived as the front vowel [ε], as in “get”. Stimuli may be obtained from the authors by email request.

### Procedure

Participants lay passively while recordings were acquired via 157-channel whole-head axial gradiometer MEG system (KIT Japan). The signal was sampled from DC to 500Hz with an online 200Hz low-pass filter and 60Hz notch filter. Earphones delivered the auditory stimuli binaurally at a comfortable listening level for the participants, approximately 74dB. Auditory stimuli were presented in two 15-minute blocks. Each participant heard two different intervals in an oddball paradigm: a series of standards with interleaved infrequent deviants. The number of standards in a row varied from 4–7 and which interval served as the deviant switched across the two blocks. The ISI varied randomly between 500–1000 ms. Participants heard 771 standards and 129 deviants in each block. Each participant heard only two intervals, and the type (vowel, sine, piano) was held constant within participants (i.e. a participant heard either V12 and V11, or S12 and S24, etc.) Thus, this experiment has a between-participants design for the contrasts of interest, although each participant contributed data for both hemispheres.

### Processing

Data were noise-reduced offline [20]. The root-mean-square average of the 10 strongest source and sink channels over auditory cortex in each hemisphere were selected from an auditory pretest and used for analyses of the MMN. Since we did not have a theory-driven hypothesis about specific sources for the MMN to our stimuli, no source localization was performed. The standard responses were subtracted from the deviant responses to get the MMN values. The set of strongest channels and the latency of the MMN response differs sufficiently across participants such that examining the grand average MMN responses (as is often done in EEG-ERP studies) is not informative due to the lack of spatio-temporal registration across the participants. Therefore, we then measured the average amplitude of the MMN and the peak latency in the window from 150–250 ms for each participant and condition in each hemisphere. The obtained amplitude and latency responses were then analyzed statistically.

## Results

Statistical analyses were conducted on each hemisphere separately because the cortical sources in each hemisphere are generated separately; hemisphere was a within-subjects variable. Linear mixed-effects models were fit for the MMN amplitude, with participant as a random effect. The fixed effects were type (piano, sine, vowel), compared intervals (12–11, 12–24) and block-order (whether 12 semitones was standard or deviant in block one), and the fixed effect interactions. In both hemispheres the main findings matched the experimental predictions: the only statistically significant effect was the interaction of type and interval (LH: F (2,30)  = 5.70, p<0.008, RH: F (2,30)  = 4.83, p<0.015). All other main effects and interactions were non-significant (p>0.05). The lack of a block-order effect suggests that the MMNs in these conditions were symmetric (that is, a dissonant interval is just as unexpected in a series of consonant intervals as the reverse).

**Figure 3:**
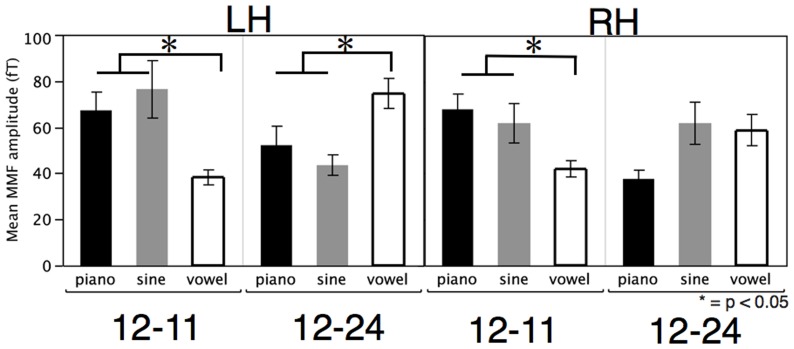
Mean mismatch field amplitudes in femtoTesla for left hemisphere (left) and right hemisphere (right) for each sound type (piano (black), sine wave (grey), vowel (white)) and interval contrast (12–11, 12–24). Asterisks indicate significant differences (p<.05) for the planned comparison between vowels and the aggregated response for piano and sine-waves.

Planned comparisons (vowels versus the aggregated sine wave and piano responses) were significant for the 12–24 contrast in both hemispheres (LH: F (1,28)  = 5.89, p<0.022, RH: F (1,29)  = 5.67, p<0.024). The 12–11 contrast was significant in the left hemisphere (F (1,30)  = 4.79, p<0.037) but not in the right hemisphere (F (1,30)  = 0.97, p>0.3), due to the strong response for the sine wave in both contrasts in the right hemisphere. Figure 3 shows the mean MMN amplitudes for the type and interval interactions in the left and right hemispheres. Linear mixed-effects models with the same design were also fit for MMN field latency. No main effects or interactions were found in the left hemisphere (all p>0.05). In the right hemisphere a main effect was found for type (F (2, 29)  = 0.32, p<0.0004). All other main effects and interactions were non-significant (p>0.05). Since we had no experimental hypotheses regarding latency, post-hoc tests (Tukey-Kramer Honestly Significant Differences, α = 0.05) were run, and show that the vowel latency was shorter than the others, but that there was no difference between the piano and sine wave latencies.

## Discussion

Overall, our predictions were borne out: the MMN amplitude mirrors the experiential intuition that the 12–11 case is closer in language-space and further in musical-space, with the 12–24 case patterning in the exact opposite manner. This evidence seems particularly strong given that participants across conditions (12–11 vs. 12–24) were hearing the exact same stimulus for the 12 semitones, with only its deviant context interval (24 or 11) changing, and that the frequencies of every type of stimulus were constant across content domains, though the spectral detail of vowels, piano tones, and sine waves differs (see Figure 2).

To address our secondary question, i.e. to what degree sine waves are really analogous to more complex naturalistic stimuli, we have some evidence suggesting complex musical stimuli (e.g. piano tones) are a better contrast to language than pure tones. This is underscored by our result that in the right hemisphere the sine wave MMN has a large response in both the 12–11 and 12–24 conditions, suggesting that such simple stimuli highlight any existing stimuli differences (see Fig. 2). Put otherwise, the piano tones and the sine wave stimuli elicit different response patterns: the piano tones pattern consistently opposite from the vowels, showing a stronger MMN in the 12–24 condition than in the 12–11 condition across both hemispheres. The sine wave amplitudes only show this pattern in the left hemisphere; in the right hemisphere, the sine wave tones elicit robust MMNs for both contrasts. This pattern of results, in turn, may suggest that sine waves are not good stand-ins for ‘musical’ stimuli, and that the better (more ecologically valid) comparison is between the piano intervals and the vowels.

While our results did not set out to test an integrated processing account, our findings lend some credence to the view that at least for the MMN response, there is some separate processing across language and music. Similarly, our findings suggest that context (as created by MMN standard stimuli) affects perception (of the deviant stimuli) in both musical and linguistic contexts, insofar as the electrophysiological MMN difference to these types of stimuli is demonstrably context- and domain-dependent in our results. A detailed account of the exact nature of the cognitive and neural processing of this difference still needs to be developed. As it is not possible to fully match the timbre between the piano and vowels (as then they would sound identical), the timbre differences certainly contribute to the perceived and measured differences. However, given the findings in [21], we do not believe that the differences in adjacent harmonic amplitudes between the piano and the vowels (see Fig. 2) are sufficient to, by themselves, invert the similarity calculation between the 12–11 and 12–24 intervals. Our conclusion is that the timbre differences are used by listeners to identify the content domain and then the appropriate similarity metric is chosen and applied. One potential test of this hypothesis would be to conduct an MMN study where octave-interval vowel and piano sounds are contrasted. We predict that the change in timbre should be at least as detectable for listeners as the changes in intervals were in this experiment.

Previous work suggests differentiated roles for the right and left auditory cortices, with the left hemisphere showing more specialization for linguistic/temporal information while the right hemisphere shows more specialization for musical/spectral information [15], [22]. However, our findings on the whole do not support such a stark contrast. It is possible that the MMN elicited by our stimuli is simply insensitive to underlying hemispheric differences.

Finally, one goal of understanding auditory processing is to create a unified account across content domains. To this end, future studies may try to combine language and music in a within-participants design. We find the recent neurobiological model of McLachlan and Wilson [23] to be potentially informative for these purposes.

In conclusion, we found a top-down influence of cognitive domain on the low-level auditory processing as indexed by the MMN. Musical sounds (in particular piano tones) elicited larger responses to the 12–11 contrast, in line with the greater dissonance of that interval comparison. Conversely, vowels elicited larger responses to the 12–24 contrast, in line with the perceived vowel difference. Thus, the low-level processing of acoustic information is influenced by the expectations induced by the cognitive domain of the sounds, suggesting that different perceptual distance metrics influence early auditory processing.
